# High Power Spark Delivery System Using Hollow Core Kagome Lattice Fibers

**DOI:** 10.3390/ma7085700

**Published:** 2014-08-07

**Authors:** Ciprian Dumitrache, Jordan Rath, Azer P. Yalin

**Affiliations:** 1 Department of Mechanical Engineering, Colorado State University, Fort Collins, CO 80523, USA; E-Mail: ciprian@rams.colostate.edu; 2 Seaforth LLC, Fort Collins, CO 80523, USA; E-Mail: jordanlrath@gmail.com

**Keywords:** fibers, laser-ignition, photonic crystal fiber, kagome, lasers

## Abstract

This study examines the use of the recently developed hollow core kagome lattice fibers for delivery of high power laser pulses. Compared to other photonic crystal fibers (PCFs), the hollow core kagome fibers have larger core diameter (~50 µm), which allows for higher energy coupling in the fiber while also maintaining high beam quality at the output (*M*^2^ = 1.25). We have conducted a study of the maximum deliverable energy versus laser pulse duration using a Nd:YAG laser at 1064 nm. Pulse energies as high as 30 mJ were transmitted for 30 ns pulse durations. This represents, to our knowledge; the highest laser pulse energy delivered using PCFs. Two fiber damage mechanisms were identified as damage at the fiber input and damage within the bulk of the fiber. Finally, we have demonstrated fiber delivered laser ignition on a single-cylinder gasoline direct injection engine.

## 1. Introduction

Despite nearly 40 years of laser ignition research [1], the technology for safe, reliable and affordable delivery systems is not yet available. Our present research aim is to test and develop an alternative approach for fiber optic delivery of laser pulses (to the engine) using recently developed hollow core kagome lattice photonic crystal fibers (PCFs). The first essential requirement for our approach is to deliver laser pulses that exceed the minimum ignition energy threshold (~15 mJ per pulse for a typical gas engine) [2,3,4,5]. A second requirement is to reliably transmit high power pulses with sufficient beam quality (low *M*^2^) such that refocusing at the output will allow for reaching intensities that lead to be optical breakdown (~300 GW·cm^−2^) [6,7,8,9].

Historically, several fiber approaches have been tested for high power pulse delivery for laser ignition including step-index fibers, hollow core fibers, photonic crystal and bandgap fibers. Coated hollow fibers have been demonstrated for spark delivery and laser ignition of a gas engine in the past [10,11,12,13]. Hollow core fibers are flexible and have typical inner (hollow) diameters of 500–1000 μm and lengths of several meters. The maximum temperature the fibers can withstand is ~500 K. The main advantage of the hollow core fibers lies in their improved output beam quality for a given core diameter when compared to solid core silica fibers. However, bending this type of fiber leads to fast deterioration in output beam quality and significant transmission losses. In one of the authors past work it was shown that, for a radius of curvature of 1 m, sparking is no longer achievable in air at atmospheric pressure [10].

Solid core step index silica fibers are potentially an attractive solution for laser ignition due to their low cost and commercial availability [14,15,16]; however, high demagnification of the output light is required in order to achieve optical breakdown in air before onset of fiber damage. The low beam quality at the fiber output, an intrinsic property of the large core multimode step-index silica fibers, makes the demagnification requirements very difficult to meet. Progress has been made with large clad step index silica fibers (clad-diameter to core-diameter exceeding ~1.1) [17,18]. The larger clad can suppress the higher order modes thus allowing better beam quality at the fiber output. Reliable ignition (100% probability with no misfires) was demonstrated in the past by our research group using fibers with a 400 µm core diameter and a 720 µm cladding through which11 mJ pulses at 25 ns duration were transmitted [17]. Nonetheless, large clad step index silica fibers are prone to vibration, bending losses, and effects of stress.

Photonic crystal and photonic bandgap fibers (PBG) employ a periodic hole structure within the silica fiber material in order to modify the refractive index in such a way as to promote efficient light guiding and single mode operation [19,20]. Nonetheless, delivery of high power pulses remains challenging and until very recently they were not suitable for ignition applications. Al-Janabi reported the use of hollow core PCFs for ignition of a methane-air mixture [21]. However, the low energy transmitted through the fiber before the onset of fiber damage (~0.15 mJ) was impractical for reliable ignition in most combustion devices. Research by Tauer *et al.* has examined PBG guiding hollow core photonic crystal fibers (HC-PCFs) [22]. The small core diameter of the PBG guiding fibers (on the order of 10 microns) and the relatively high power overlap between the hollow core and the silica structure limited the pulse energy to ~1 mJ.

The present investigation examines recently developed hollow core kagome lattice fibers which are a special kind of hypocycloid-shaped HC-PCF. Beaudou *et al.* showed ignition of a butane mixture at stoichiometric conditions using the kagome PCFs. They were able to transmit 4 mJ pulses of 10 ns duration [23]. These fibers provide light guiding via inhibited coupling (IC) between the core and the cladding modes [24,25]. Specifically, the fibers employ a hypocycloid-shaped internal structure that enhances the IC through a significant decrease in the spatial overlap between the slow-varying transverse-phase core modes and the fast-varying transverse-phase modes of the region surrounding the core. The fibers maintain single mode operation with relatively large core dimensions (mode field diameter ~50 µm) which enables transmission of high power pulses without damage.

Our current work provides a more extensive examination of kagome fibers for laser ignition. We analyze the capability of the fiber delivery system to transmit high power laser pulses as well as the mechanisms that lead to fiber damage. In Section 2, we present the experimental setup developed for testing the hollow core kagome fibers and, in Section 3, we discuss results obtained from testing the fibers. Section 4 contains concluding remarks and outlines future research plans.

## 2. Experimental Setup

### 2.1. Bench-Top Testing

**Figure 1:**
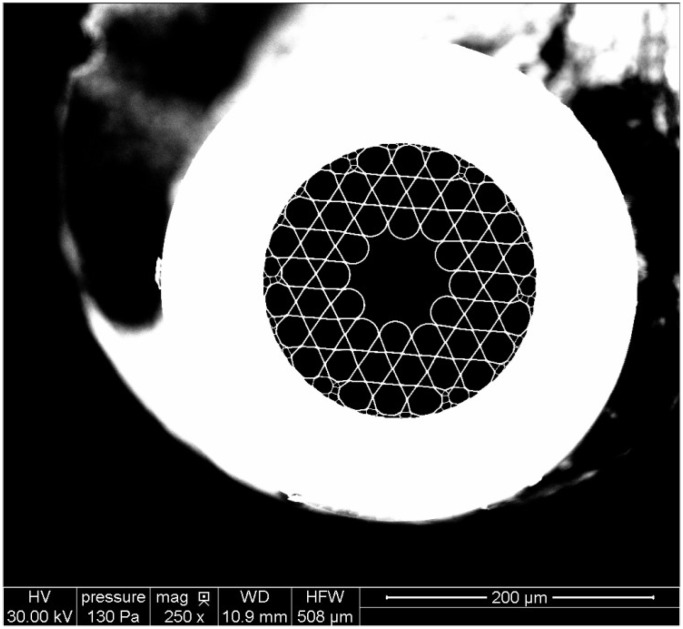
Backscattered SEM image of the kagome type hollow core photonic crystal fiber (HC-PCF) used in laser ignition experiments (Image is courtesy of GloPhotonics).

**Figure 2:**
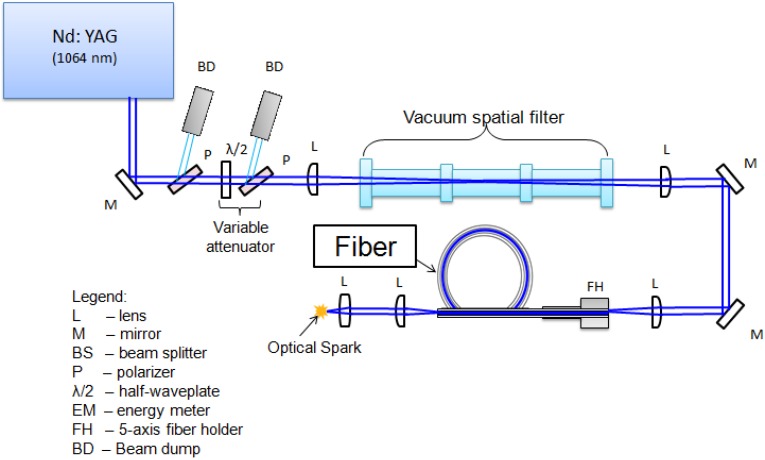
Experimental setup used for fiber testing.

Fiber characterization was initially performed in order to determine the beam quality and the maximum pulse energy that can be transmitted through the fiber. The fiber we have tested is very similar to that discussed above and previously tested by Beaudou *et al.* [23]. A backscattered scanning electron microscope (SEM) image of the fiber used in experiments is shown in Figure 1. Figure 2 outlines the setup used for fiber testing. A Q-switched, pulsed, Nd:YAG laser (Continuum Powerlite 8010, San Jose, CA, USA) at 1064 nm with a beam quality of *M*^2^ = 2 was used as the light source. The beam passed through a variable attenuator consisting of two polarizers and a half-waveplate that were used to set the beam polarization and vary the laser power. Next, the laser beam was passed through a vacuum spatial filter to improve beam-quality prior to the fiber input. As with other single-mode fibers, the input beam should be near single mode, *i.e.*, with *M*^2^ close to unity, since higher order modes do not propagate yet still tend to cause damage. The spatial filter consisted of a plano-convex focusing lens (*f* = 2000 mm) used to focus the light through a pinhole (diameter of 400 µm) after which the light was re-collimated with a second lens to a diameter of ~6 mm. As a result of the beam passing through the spatial filter, the higher order TEM modes were suppressed and the emerging beam had an improved beam quality, *M*^2^ = 1.18. The beam was then steered using a pair of mirrors to fiber launch location. Several launch configurations were tested with the best results obtained using a 125 mm focal length plano-convex lens. The numerical aperture at the launch was measured to be ~0.013 and the focused diameter at the fiber input was 41 µm. To enable alignment, the fiber tip was mounted on a 5-axis translation stage. For spark formation examination, the light exiting the fiber was tightly focused with an aspheric lens of focal length *f* = 10 mm.

### 2.2. Engine Testing

In contrast with our bench top testing presented in Section 2.1, the overall laser ignition system for engines demonstration includes the laser, the launch optics together with an optical spark plug built to interface with the engine’s cylinder.

**Figure 3:**
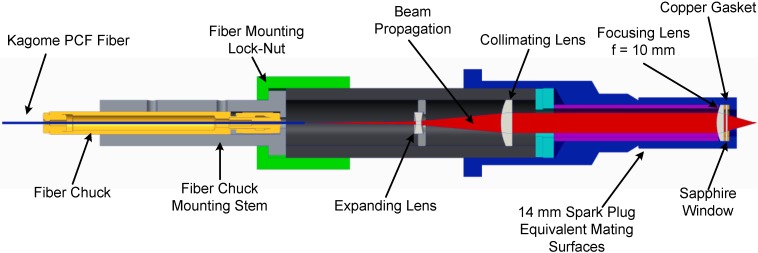
Assembly view of the optical spark plug design.

An optical spark plug was designed to mate with the 14 mm spark plug port on the engine through which the light would be directed and focused into the engine for sparking. The optical plug contains the fiber output as well as optics to shape and focus the beam. Figure 3 shows a cross-sectional image of the optical spark plug used in the engine testing. The beam was collimated after exiting the fiber using a negative lens (Thorlabs, Newton, NJ, USA, LD2746-C, *f* = −6 mm) to expand the beam over a shorter distance, followed by a second lens (Thorlabs LA1289-C, *f* = 30 mm) that collimates the beam to a diameter of 5.5 mm. The beam is then directed onto a 10 mm focal length lens (LightPath, Orlando, FL, USA, Gradium GPX10-10) which focuses the beam into the engine cylinder. The Gradium lens is selected to provide a small focal spot to promote high focused intensity and spark formation. Copper gaskets on either side of a sapphire window (Edmunds 43-629, Barrington, NJ, USA) at the tip of the spark plug seal the rest of the optics away from the engine cylinder and protect the optics from the engine fluids and high pressure conditions.

## 3. Results and Discussion

### 3.1. Beam Quality

By carefully optimizing the position at fiber launch we were able to obtain a maximum power transmission through the fiber of ~85% with output beam quality factor *M*^2^ = 1.24. The *M*^2^ of the fiber output was determined using the method described by Siegman [26]. The CCD beam profiler was mounted on a *Z*-axis translation stage and measurements of the beam diameter at various distances around the beam waist were taken. Beam diameter was determined with the beam profiler using the D4σ method. The *M*^2^ was then obtained by curve fitting the data based on the following formula:

(1) $$W{\left(z\right)}^{2}=W_{0}^{2}+M^{4}\times {\left(\frac{\lambda }{\pi W_{0}}\right)}^{2}{\left(z-z_{0}\right)}^{2}$$

**Figure 4:**
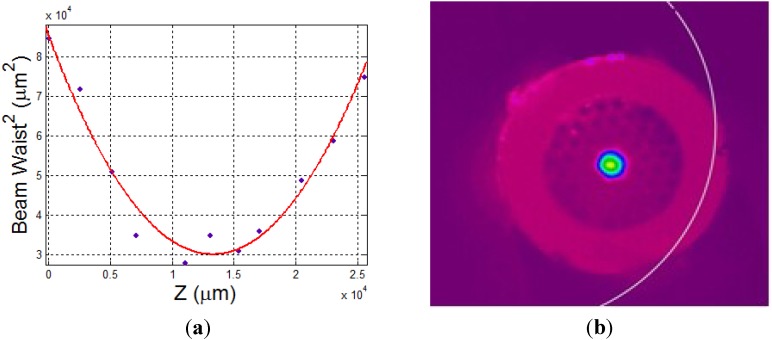
(**a**) Data curve fit for *M*^2^ measurements; (**b**) Far-field image of the fiber output.

Curved fitted data for obtaining *M*^2^ are shown in Figure 4a. Figure 4b shows the beam as it is seen through a CCD (Charge-coupled Device) camera at distance of 3 cm beyond the fiber output.

For the nominal laser pulse duration of 12 ns, the maximum output energy (prior to damage onset) was 11 mJ. To examine spark formation, the light exiting the fiber was tightly focused with an aspheric lens of focal length 10 mm. The conditions described above (for 12 ns pulse duration) could readily form a spark at the focused output.

### 3.2. High Power Transmission

**Figure 5:**
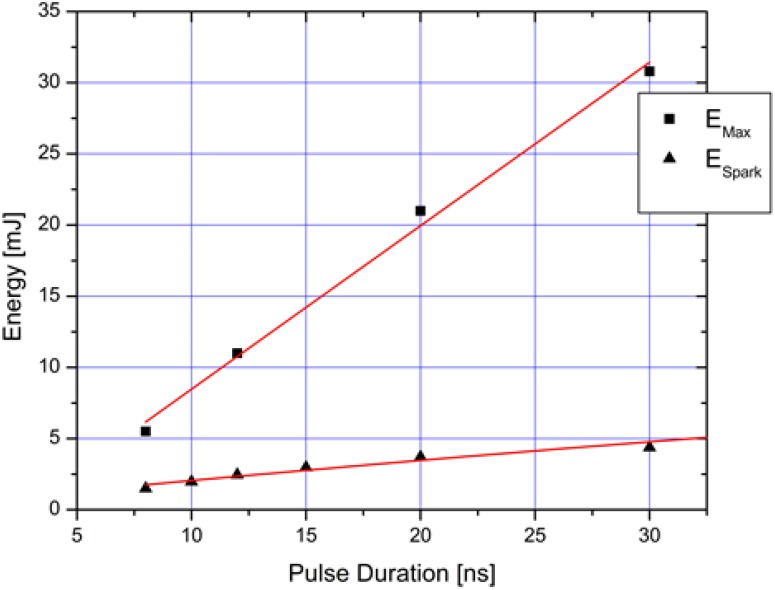
Dependence of maximum laser energy before damage (*E*_Max_) and minimum laser energy to spark at output (E_Spark_) on laser pulse duration.

In order to better understand the fiber limitations and highest energies that can be transmitted, we have varied the laser pulse duration. By increasing the duration of the pulse (at fixed energy), the peak power per pulse is reduced and, as a consequence, the light intensity at the fiber tip is also minimized. A lower intensity on the fiber tip allows more delivered energy prior to damage onset. We examine both the maximum energy that could be transmitted through the fiber, *E*_Max_, and the minimum (input) energy that was required to generate sparks (in atmospheric pressure air with 100% probability) by refocusing the fiber output, *E*_Spark_. Figure 5 shows that the amount of energy delivered by the kagome fiber varies approximately linearly with the pulse width. We were able to transmit as much as 30 mJ of energy through the fiber by employing pulse duration of 30 ns. Previous studies on laser ignition [3,4,5,8] have shown that ignition of lean fuel-air mixtures requires sparks with ~15 mJ of energy, while ignition at stoichiometric conditions requires less energy (~2 mJ). To generate sparks in air at atmospheric conditions, (*P* = 0.85 atm, *T* = 298 K in Fort Collins, CO, USA) our setup required energy of only 1.4 mJ for a 7 ns pulse. However, for this pulse duration, fiber damage occurred at 5 mJ. By stretching the pulse to 30 ns, almost 5 mJ were needed to create sparks but fiber damage occurred at much higher energies (~30 mJ). In summary, we find that increasing the pulse duration leads to an increase in the energy deliverable through the fiber while also increasing the safety operation margin. For example, we can couple six times more energy into the fiber, beyond initial spark formation, before onset of fiber damage when using a 30 ns pulse.

### 3.3. Fiber Damage Modes

**Figure 6:**
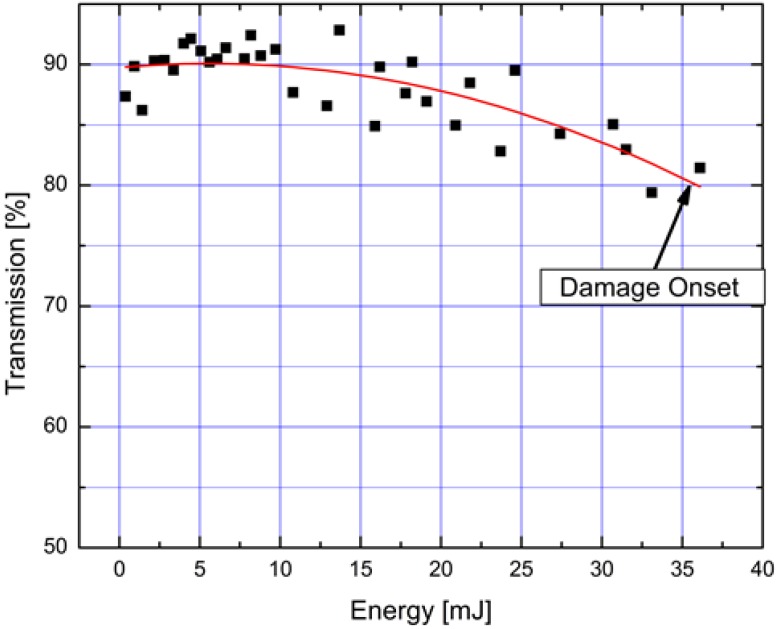
Example of fiber transmission as input energy was increased, for case where fiber fails at input tip (30 ns pulse duration).

Figure 6 shows the evolution of power transmission through the kagome fiber as the energy input was varied using the variable attenuator depicted in Figure 1. Initially the transmission stays constant when the input energy is increased; however, as the damage limit is approached, the transmission reduces. Throughout all the tests conducted in our lab, damage on the tip of the fiber appears immediately after the transmission drops below 80%. In this experiment, the damage is seen to occur on the fiber tip and it propagates inward at a rate of ~2 cm·min^−1^ if the laser is not immediately turned off. The damage results in the complete destruction of the internal structure of the fiber at the fiber tip. Nonetheless, the fiber can be re-used by cleaving the damaged end. The main cause of this type of damage is the increase intensity on silica structure of the fiber tip at launch [27,28]. In the particular case shown in Figure 6, damage occurs when the input energy is increased to 35 mJ. The use of larger core fibers that still maintain (near) single-mode output would be very enabling in this context.

**Figure 7:**
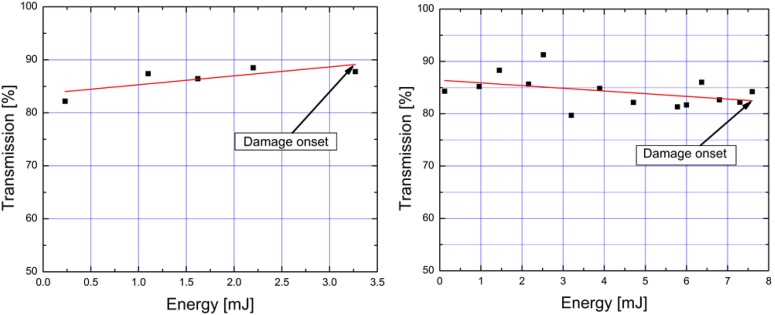
Examples of fiber transmission as input energy was increased, for cases where fiber fails within its length (12 ns pulse durations).

Damage on the inside of the fiber (within its length) was also encountered in a few occasions during testing. In Figure 7a,b, we show again the power transmission as the input energy was increased. The pulse width in both cases was 12 ns. In these cases, fiber breakdown occurred at much lower energies as compared to the maximum energy in Figure 5. This type of damage was not preceded by a decrease in energy transmission, as was the case with the damage induced by high intensities on the fiber input tip (Figure 6). We have identified several mechanisms that can be responsible for this type of damage: laser power fluctuations and beam wander, optical feedback caused by back-reflected light downstream of the fiber, or imperfections/micro-fractures inside the fiber. This type of damage was very destructive: Once the fiber failed at the weak point, the damage propagated through at a rate of ~5 cm·s^−1^, also vaporizing on its way the kagome lattice internal structure of the fiber.

### 3.4. Engine Testing

**Figure 8:**
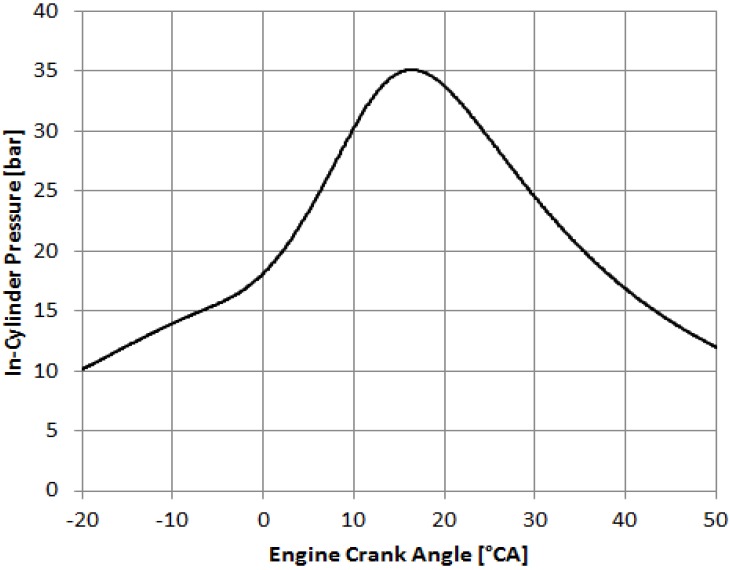
Pressure trace for a single engine cycle.

As part of our investigation, we have used the kagome fibers to conduct preliminary engine tests [29]. The tests used a Ford Single Cylinder Research Engine set up for Gasoline Direct Injection (GDI) operation with variable equivalence ratio and load condition capabilities at Argonne National Laboratory, Chicago, IL. For fiber delivery ignition tests, the engine was motored to 2400 rpm (corresponding to a ~20 Hz repetition rate) and the laser was externally triggered using a 5 V signal referenced to the engine crank angle. Initial short duration tests achieved reliable (100% probability) ignition using the kagome PCF fiber delivery system. Figure 8 shows an example pressure trace from a single combustion cycle. The engine was operated at a stoichiometric fuel-air ratio and exhibited a 5.85 bar indicated mean effective pressure (IMEP) with a coefficient of variation (COV) of 1.84%. The relatively low COV value, below 2%, indicates that the laser ignition provided stable repeatable combustion.

## 4. Conclusions

We have developed and tested a fiber delivery system for laser ignition applications using hollow core kagome lattice fibers. Using our setup, energies on the order of 30 mJ were successfully delivered through the fiber by employing a laser pulse width of 30 ns. Analyses of the energy delivered through the fiber versus pulse width show an approximately linear dependence where increase in pulse duration leads to increase of the amount of energy that can be delivered through the fiber. Preliminary results show that the fiber deliver system can reliably ignite an engine at stoichiometric conditions without misfire. In the future we plan to test our fiber delivery system for various engine operation conditions with the goal of showing long term reliable operation and ignition of lean fuel-air mixtures.
